# MAX IV Laboratory

**DOI:** 10.1140/epjp/s13360-023-04018-w

**Published:** 2023-06-05

**Authors:** Aymeric Robert, Yngve Cerenius, Pedro Fernandes Tavares, Anna Hultin Stigenberg, Olof Karis, Ann-Christine Lloyd Whelan, Caroline Runéus, Marjolein Thunnissen

**Affiliations:** MAX IV Laboratory, Lund University, BOX 118, 211 00 Lund, Sweden

## Abstract

MAX IV Laboratory is a Swedish national synchrotron radiation facility that comprises three accelerators with varying characteristics. One of the accelerators, the 3 GeV storage ring, is the world’s first fourth-generation ring and pioneered the use of the multibend achromat lattice to provide access to ultrahigh brightness X-rays. MAX IV aims to stay at the forefront of the current and future research needs of its multidisciplinary user community, principally located in the Nordic and Baltic regions. Our 16 beamlines currently offer and continue to develop modern X-ray spectroscopy, scattering, diffraction, and imaging techniques to address scientific problems of importance to society.

## Introduction

MAX IV Laboratory is a Swedish national synchrotron laboratory located in Lund (Sweden) and is hosted by Lund University [1]. It is the successor of MAX-lab,^1^ which hosted three accelerators that were in operation between 1987 and 2015, MAX I, II, and III, inaugurated in 1987, 1995, and 2007, respectively. MAX III was a testbed for some of the technological developments that paved the way for the design of MAX IV [3]. During all these years, these accelerators supported the research needs of the nuclear physics community and the ones of an ever-growing X-ray user community in Northern Europe. The user community at MAX-lab was initially based on a very strong tradition in various forms of X-ray spectroscopies in the soft X-ray regime, but with the development of new beamlines for protein crystallography, diffraction, and EXAFS in materials science, the user community grew rapidly, pointing toward a motivation for a further expansion of synchrotron capabilities toward harder X-rays in Sweden.

In April 2009, Lund University, the Swedish Research Council (VR, [4]), Region Skåne [5] and the Swedish Innovation Agency (Vinnova, [6]) decided to fund MAX IV. Construction began in 2010, and the facility was inaugurated in June 2016 [7].

MAX IV, hosted by Lund University, receives financial contributions for the construction and operation of the accelerators, beamlines, and supporting infrastructures from several funders, including the Swedish Research Council (VR), The Knut and Alice Wallenberg Foundation (KAW, [8]), the Council for Sustainable Development (Formas, [9]), Energimyndigheten [10], the Treesearch Consortium [11], the Novo Nordisk Foundation (NNF, [12]) in Denmark, the Danish Government and three Danish universities,^2^ and the Academies of Finland [16] and Estonia [17], among others. Through a special agreement, 14 Swedish universities also make direct contributions to the operations budget of MAX IV.^3^

MAX IV Laboratory consists of a 3 GeV and a 1.5 GeV storage ring and a linear accelerator that serves as an injector to both rings as well as a driver for our Short Pulse Facility. Altogether they provide the basis for our vision of being “*A world-leading synchrotron laboratory enabling world-class science for a better future*.”

Our 3 GeV storage ring, the first fourth-generation storage ring in the world [31–34], is the result of a nearly 20-year-long development process [35]. This was triggered by the pioneering works of Einfeld et al. [36], who proposed a multibend achromat (MBA) lattice layout as a means to achieve ultra-low emittance and ultra-high brightness. More than a decade of design and prototype work (including the construction of the small, 36 m circumference, MAX III storage ring as a testbed for enabling technologies) was required before the technical challenges brought about by the MBA concept could be addressed convincingly, and the actual construction of the MAX IV 3 GeV ring could be started [3].

The overarching engineering challenge in the implementation of the MBA lattice is compactness, i.e., the need to fit a large number of components into as small as possible a circumference in order to minimize the facility size and associated costs. MAX IV addressed that challenge with an integrated approach to the design of the key accelerator subsystems, namely magnets, vacuum, and radio-frequency (RF). A cornerstone of the design is the use of small-aperture, combined-function magnets [37] that allows reaching larger integrated gradients in shorter magnets and reduces the minimum required distance between consecutive magnets. In addition, these compact magnets are built as integrated units in which the bending magnet poles and quadrupole pole roots are machined out of solid iron blocks, each unit holding all the magnets of a complete cell. This design leads to excellent alignment accuracy and gives high natural vibration frequencies of the units, thus reducing the sensitivity of the magnets to floor vibrations. The compact magnet design, in turn, requires narrow low-conductance vacuum chambers [38], which necessitate distributed pumping and distributed absorption of the heat load from synchrotron radiation. The heat load problem is dealt with by choosing copper as the chamber material and providing water cooling along the extended region over which the synchrotron radiation heat is deposited, while distributed pumping is provided by non-evaporable getter (NEG) coating of the chamber’s inner surface [39]. Finally, the RF system [40] uses capacitive-loaded normal conducting cavities operating at 100 MHz, which allows a large bucket height with relatively low RF voltage and power to be achieved, enabling the use of standard high-efficiency, low-cost solid-state RF transmitters largely used in telecommunications. Moreover, through the use of passively operated third-harmonic cavities (Landau cavities), an elongation of the electron bunches by up to a factor of five can be achieved. This is essential to keep the heat load from beam-induced fields on the narrow vacuum chamber down to acceptable levels and prevents transverse beam blow up due to intrabeam scattering. Finally, the elongated bunches also possess a large spread of synchrotron frequencies which are key to maintaining longitudinal stability and preserving the brightness of high-order undulator peaks.

Sirius at the Brazilian Synchrotron Light Laboratory (LNLS, Campinas, Brazil, [41]) and the European Synchrotron Radiation Facility’s Extremely Brilliant Source (ESRF-EBS, Grenoble, France, [42]) were the first facilities to follow MAX IV and implement variants of the MBA lattice.

MAX IV, with its 16 beamlines currently in operation, provides access to modern X-ray analytical tools with X-ray spectroscopies, scattering and diffraction, and imaging techniques. We cater to a multidisciplinary user community that addresses science questions of relevance for society in many areas, such as health and medicine, environmental challenges, quantum and advanced materials, energy technologies and materials, to name a few.

Every year, MAX IV welcomes more than a thousand users from academia and industry. As the last beamlines were just built and other beamlines are reaching a certain level of maturity, we expect this number to grow over the coming years. In 2021, more than 75% of our users were principally originating from Swedish universities and from institutions from the Nordic (i.e., Denmark, Finland, Iceland, Norway, Sweden) and Baltic (i.e., Estonia, Latvia, Lithuania) regions. MAX IV is a member of the League of European Accelerator-based Photon Sources (LEAPS) [43].

Access to our beamlines is open to academia, research institutes, industry, and government agencies worldwide. Access is granted based on the peer-reviewed evaluation of the scientific merit and technical feasibility of science proposals submitted twice a year. Other access modes are and will also be available, such as Teaching and Education proposals, Rapid Access, and Long-Term proposals. Access is without charge for users who intend to publish their results open access.

We also offer a proprietary access program that can be of interest to industry, institutes, and private/public organizations performing commissioned research funded by industry. This service is offered for a fee that provides fast access to our beamlines and full confidentiality and IP control over the produced data. Industry users are strongly encouraged to contact our Industry Relation Office for more details [44]. Currently, the industry use of MAX IV represents about 1–2% of the total amount of beamtime on all our instruments. We believe that there are growth opportunities that could reach up to a maximum of 5% on average for the entire laboratory.

## Present status and scientific highlights

MAX IV consists of a 3 GeV storage ring, a 1.5 GeV storage ring, and a Short Pulse Facility. The 3  GeV storage ring (R3) with a circumference of 528 m is geared principally toward hard X-ray users, while the 1.5 GeV storage ring (R1), 96 m circumference, serves UV and soft X-ray users. The Short Pulse Facility (SPF) serves the needs of the ultrafast science community. Some of the relevant parameters of each accelerator are provided in Table.1.

**Table 1:** Nominal parameters of the three MAX IV accelerators: The 3 GeV ring (R3), the 1.5 GeV ring (R1) and the linear accelerator (Linac) that serves as injector to the rings and as a driver to the Short Pulse Facility. Here, we show the Linac parameters when running as a driver to the SPF. MAX IV currently has 16 beamlines in operation. The storage rings may house an additional 14 beamlines and the Short Pulse Facility can accommodate two more

Parameter	Accelerator		
	Unit	R3	R1
Energy	GeV	3	1.5
Circumference	m	528	96
Achromat type		7BA	2BA
Straight sections (avail. for ID)		20 (19)	12 (10)
Straight section length$^1$	m	4.6	3.5
Maximum current	mA	500	500
RMS bunch length	ps	200	200
Horizontal emittance	pm rad	328$^2$	6000
Vertical emittance	pm rad	8	60
Energy spread	$10^{-3}$	0.77	0.75
Beam lifetime	h	10	10
Beamlines in operation		10	5

Parameter	Unit		Linac
Energy	GeV		3
Length	m		$\sim$300
Maximum bunch charge	pC		100
Bunch length	fs		100
Maximum repetition rate	Hz		100
Beamlines in operation			1

1 Typical full straight section length. Some of them can occasionally be shorter. The length available for magnet assembly of insertion devices varies depending on the ID design

2 Depending on the number and settings of the insertion devices, the horizontal emittance may be further reduced

The number of beamlines in operation for our user science program has gradually increased from 2 in 2016 to 16 in 2023. The two last additions are the MicroMAX and ForMAX beamlines [34] that will mainly contribute to life science and research on wood-based materials, respectively. The beamlines that MAX IV currently operates are distributed as follows: 5 soft X-ray beamlines on R1 (FinEstBeAMS, Bloch, MaxPEEM, FlexPES, and SPECIES), 3 soft X-ray beamlines (SoftiMAX, VERITAS, and HIPPIE) and 7 hard X-ray beamlines (BioMAX, NanoMAX, CoSAXS, DanMAX, BALDER, ForMAX, MicroMAX) on R3, and 1 hard X-ray beamline at SPF (FemtoMAX).

**Fig. 1:**
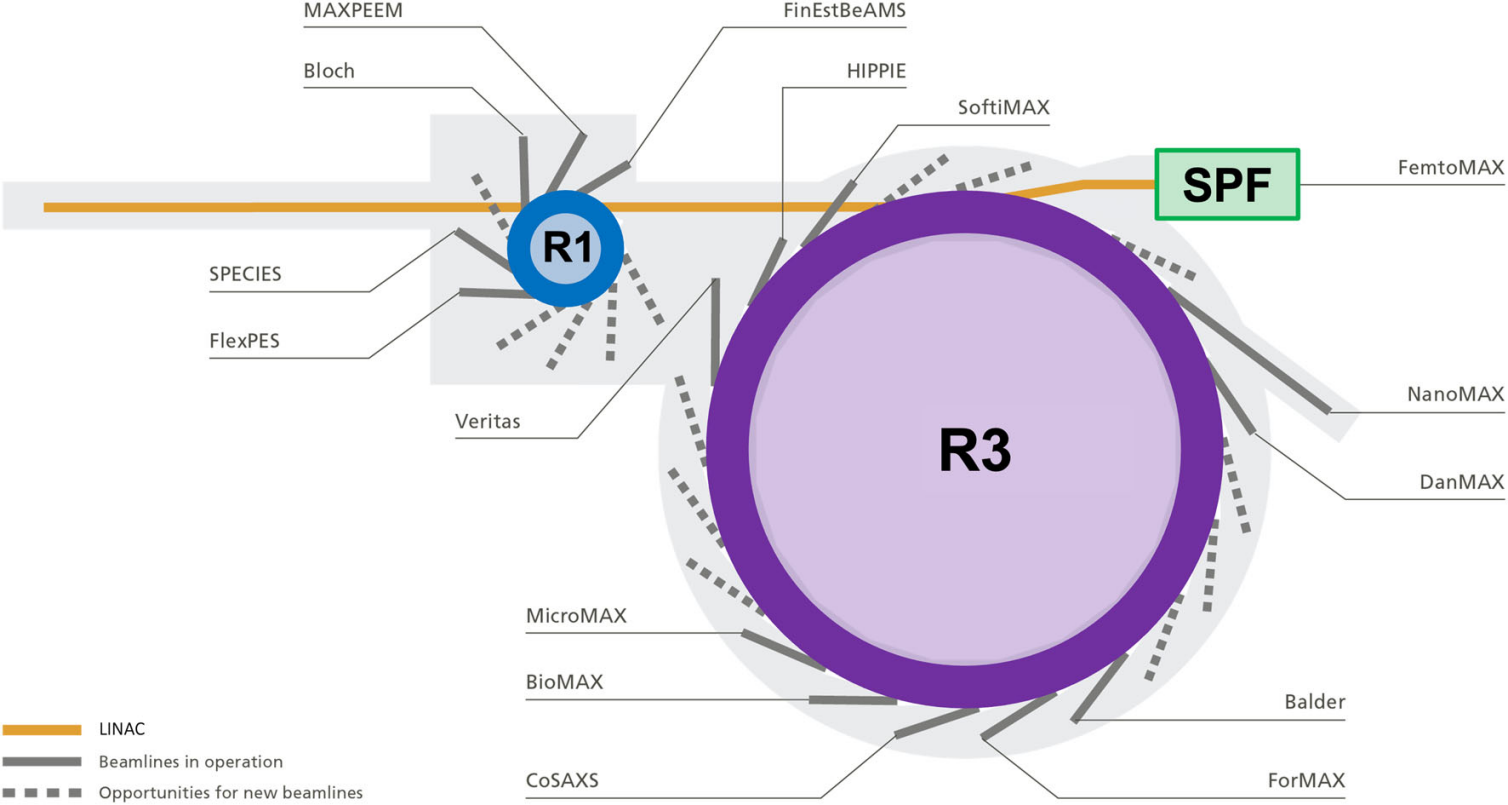
Schematic layout of MAX IV and its 16 beamlines on their respective accelerators R1, R3, and SPF. Relevant parameters from each accelerator are provided in Table 1. Details of the X-ray spectroscopy, diffraction and scattering, and imaging techniques available at each beamline are provided in Table 2. The dashed lines illustrate the location of possible future beamlines

The location of each beamline on their respective accelerator (R1, R3, or SPF) is shown in Fig. 1. MAX IV has the opportunity for up to 14 additional beamlines to be placed at the straight sections of its storage rings: 5 soft X-ray beamlines on R1, and 9 soft or hard X-ray beamlines on R3, as indicated by the dashed lines in Fig. 1. R1, an ideal source for soft X-ray beamlines, can also be an adequate source for developing an infrared program.

**Fig. 2:**
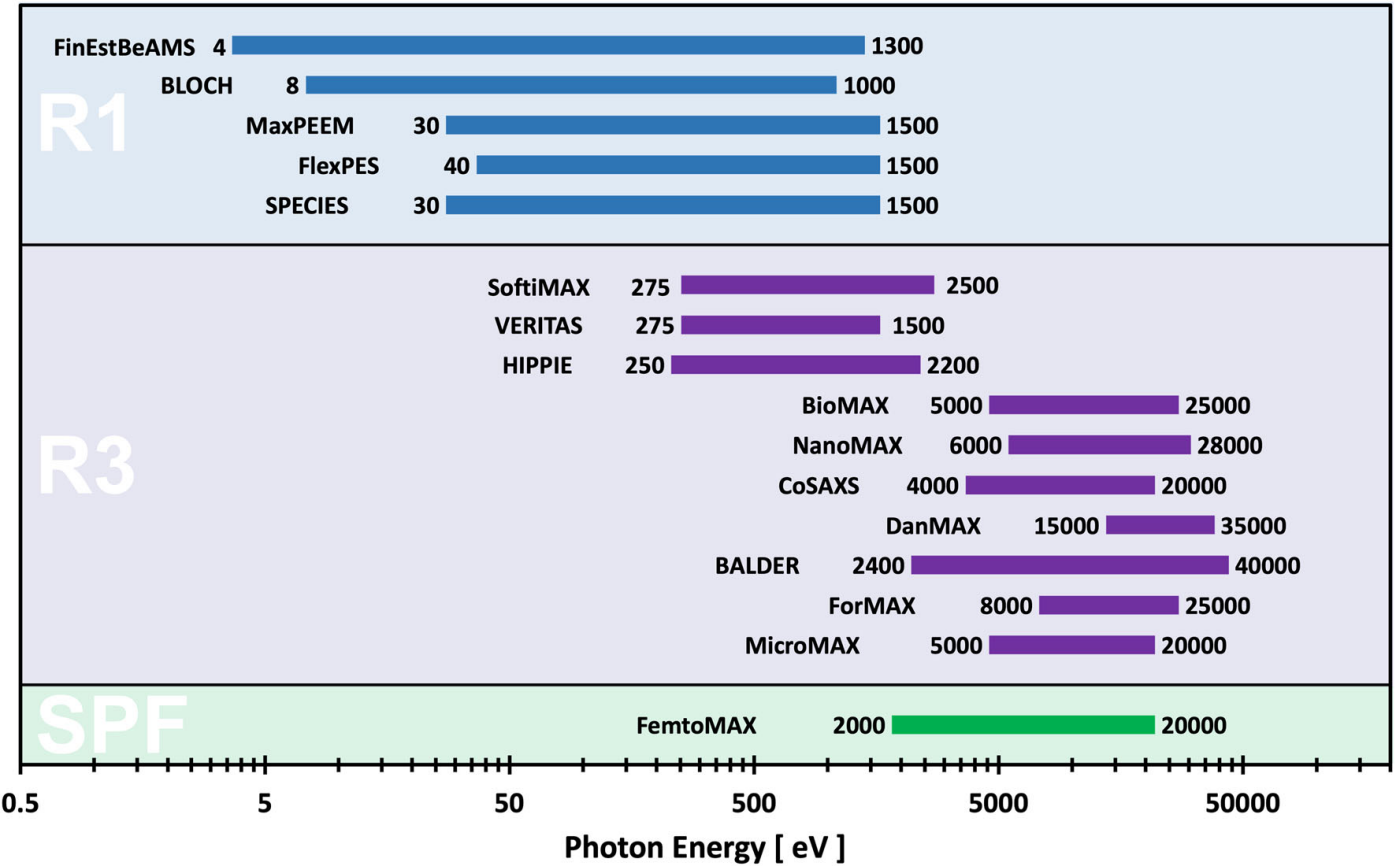
X-ray photon energy range for the 16 MAX IV beamlines on the R1, R3 storage rings, and at the Short Pulse Facility (SPF)

Our beamline portfolio provides access to modern soft and hard X-ray analytical tools with spectroscopy, scattering and diffraction, and imaging techniques as described in detail in Table 2. A reference to a relevant beamline publication is also provided in the table, if available. The table also lists if the core techniques of the beamlines focus on detecting photoelectron, visible, or X-ray photons. Our beamline portfolio covers a broad range of X-ray photon energies from 4 eV up to 40 keV, as detailed in Fig. 2. We note that, whereas the 3 GeV storage ring hosts all hard X-ray beamlines, it also comprises three soft X-ray beamlines, that are nearly diffraction limited on the lower end of their photon energy range.

All beamlines on R3 take advantage of the uniquely small emittance of this source in several ways. This translates into unprecedented opportunities for being at the forefront and developing X-ray techniques that benefit from X-ray beams with small divergence, small beam size, a large number of photons per energy bandwidth unit, and a large fraction of coherent X-rays.

The scientific areas that MAX IV contributes to are diverse and broad. Below are some examples that are detailed in our 2021 Science Highlights [45]:

- *Advanced materials* Investigate with scanning X-ray diffraction microscopy the relationship between defects and magnetic domains in antiferromagnets [46], but also to get a better understanding of the relationship between the size of nanoparticles and their atomic structure with pair distribution functions [47].
- *Environmental Sciences* Intend to better understand Martian salt chemistry by probing the chemical details of solvated surfaces, including salts, and how they play a role in gas formation and secondary aerosol particle formation [48].
- *Catalysis* Providing new insight into the dynamics of a catalytic reaction in ambient conditions with a new experimental approach of event-averaging time-resolved Ambient Pressure X-ray Photoelectron Spectroscopy [49].
- *Health and medicine* Better understanding of amyloids using correlative imaging to assess the structural and chemical information within a single cell of neurons [50].
- *Structural Biology* Uncovering an inhibitor for SARS-CoV-2 with a promising pharmacological profile [51] and how plants signal and control interactions with symbiotic bacteria [52].
- *Energy materials* Boosting processing technology for metal-halide solar cells by slot die coating [53]; but also better understanding the degradation of copper electrodes [54].

**Table 2:** Description of the available spectroscopy, diffraction and scattering, and imaging techniques for each beamline. Also indicated is the principal detection from each beamline, whereas visible light (vis), photoelectron (e-), or X-rays. The last column provides a link to a beamline article, if available

Beamline	Techniques	Detection	Ref.
*R1–1.5 GeV storage ring*			
FinEstBeAMS	XPS, PLS$^1$, UV-VUV spectro. (PEPICO$^2$, TOFMS$^3$)	e-, vis	[55]
Bloch	ARPES, spin-ARPES, STM, XPS	e-	–
MaxPEEM	SPELEEM$^4$, $\mu$-XPS, $\mu$-XAS, nano-ARPES	e-	[56]
FlexPES	XPS, XAS, PEPICO	e-	–
SPECIES	AP-XPS$^5$, RIXS	e-	[57]
*R3–3 GeV storage ring*			
SoftiMAX	STXM, CXDI, Imaging	X-ray	[58, 59]
VERITAS	RIXS	e-	–
HIPPIE	AP-XPS$^5$	e-	[60]
BioMAX	High-throughput MX, MAD, SAD, XRF	X-ray	[61]
NanoMAX	Imaging, $\mu$-XRF,$\mu$-XRD, Tomogr	X-ray	[62]
CoSAXS	SAXS, WAXS, Bio-SAXS, XPCS	X-ray	[63]
DanMAX	XRD,PRD, PDF, TS, $\mu$-XRF,$\mu$-XRD, Tomogr	X-ray	–
BALDER	Q-EXAFS,XAS, XRF, XES, XRD	X-ray	[64]
ForMAX	SAXS, WAXS, Imaging, Tomogr	X-ray	–
MicroMAX	MX, Serial-MX	X-ray	–
*SPF–Short Pulse Facility*			
FemtoMAX	Pump-Probe XRD & XRF	X-ray	[65]

^1^Photo Luminescence Spectroscopy

^2^PhotoElectron Photo-Ion Coincidence Spectroscopy

^3^Time Of Flight Mass Spectroscopy

^4^Spectroscopic PhotoElectron Low Energy Electron Microscope

^5^Ambient Pressure PhotoElectron Spectroscopy

## Technological developments and upgrade plans

In this section, we describe some of the developments and upgrades that we envision for both the accelerators and beamlines of MAX IV. Together they will be key in successfully ensuring MAX IV’s ability to respond to the research needs of its user community.

### Accelerators

A roadmap of accelerator developments has been defined to maintain the MAX IV sources internationally competitive while many other facilities worldwide follow suit and develop their variants of the MBA lattice. This roadmap considers all three accelerators that make up MAX IV Laboratory. It includes shorter-term (i.e., 3–5 years) incremental improvements and longer-term (i.e., 5–10), radical, game-changing proposals. The shorter-term initiatives aim to meet the user community’s immediate needs while establishing critical enabling technologies that set the stage for longer-term upgrades. In this way, the roadmap forms a coherent set of activities on multiple time scales making the most cost-effective use of synergies between the various efforts. Some of them are described below.

Preliminary design studies [66] indicate the possibility of achieving reductions by a factor of 1.2$-$1.5 of the stored beam emittance in the 3 GeV ring with minimal hardware changes. The main challenge is demonstrating efficient injection into these configurations. These changes would lead to up to $\sim$40% brightness increase for 10 keV photons. This increase would be in addition to the expected brightness improvements resulting from the emittance reductions associated with adding insertion devices to the storage ring. The first experimental studies were conducted in 2021 [67].

Emittance reduction by a factor of more than two and a corresponding increase in brightness is also conceivable [68] through moderate changes in the magnet lattice. Together with changes to the injection scheme, these could enable the use of advanced insertion devices with a reduced horizontal aperture, such as the APPLE-X undulators currently being prototyped [69] in connection to the Soft X-ray Laser project [70].

Achieving the diffraction limit for $\sim$10 keV X-rays requires reaching $\sim$10 pm rad emittance. Preliminary design studies [71, 72] show that this is possible even if challenging. Such an upgrade requires completely replacing the magnet lattice and vacuum system but would use most of the existing infrastructure, including the RF systems. Various already ongoing technical developments will be relevant to achieving such performance improvements: e.g., (i) the injection scheme would benefit from the developments of fast kickers, and (ii) the implementation of ultra-long bunches will be crucial to maintain the ultra-low emittance despite the effects of intrabeam scattering in high-intensity bunches.

Long bunches are crucial in achieving ultra-low emittance and, consequently, ultrahigh brightness. Lengthening factors up to around five are obtained today in the MAX IV rings using passive third-harmonic cavities. Theoretical studies^4^ indicate that achieving even larger factors by combining third and fifth-harmonic cavities is possible. This would provide enhanced stability and help reduce the impact of intrabeam scattering at the existing 3 GeV ring and, at the same time, pave the way for future projects aiming at even smaller emittances.

Transparent top-up injection has been successfully implemented in the 3 GeV ring [73], where a world-record low perturbation to the stored beam was achieved using a Multipole Injection Kicker (MIK) developed in collaboration with Synchrotron SOLEIL [74]. A prototype of a similar device was recently installed and successfully commissioned in the 1.5 GeV ring; the main challenge being the reduced pulse length required due to the smaller circumference [75]. Besides the immediate benefit to all beamlines in the 1.5 GeV ring, the required development of high-voltage fast pulser technologies is closely connected to the future needs of an upgraded 3 GeV ring based on on-axis injection schemes and its capabilities of hosting advanced insertion devices.

To satisfy the needs of scientific experiments requiring customized time structures of the synchrotron radiation, the 1.5 GeV ring is today run in “single-bunch” mode for a few weeks every year. While this mode enables exciting science, it also reduces the total intensity to the detriment of other experiments, which are impossible under those circumstances. One scheme that could potentially allow the simultaneous use of the source by both communities was pioneered at BESSY [76] with Transverse Resonant Island Buckets (TRIBs). This scheme has already been successfully tested in demonstration experiments at MAX IV [77]. Even though this scheme only has minor requirements on new hardware developments, its feasibility under routine delivery remains to be demonstrated: significant challenges include running at high currents and maintaining stable transverse tunes while insertion device gaps and phases change. Other schemes requiring the development of advanced fast, high repetition rate kickers capable of putting a single bunch onto a displaced orbit will be explored in a second phase.

Despite their outstanding transverse emittance and corresponding brightness and coherence, the X-ray pulses produced by the 3 GeV ring are relatively long. Long bunches are, in fact, a prerequisite for achieving extremely low emittance. As a result, science involving physical phenomena happening at the sub 100 fs timescale remains out of reach. The only demonstrated mechanism to produce extremely bright and, at the same time, ultrafast X-ray pulses is the free-electron laser. Our linear accelerator is prepared from its design to operate as a driver of a free-electron laser and has, in particular, bunch compressors that can generate extremely short electron bunches. A conceptual design study for a soft X-ray free-electron laser driven by the MAX IV linac was conducted, and the resulting Conceptual Design Report (CDR) was published in spring 2021 [70, 78].

### Beamlines

In 2023, MAX IV operates 16 beamlines, some of which have been accepting users for more than five years, while others, like ForMAX [34] or MicroMAX just finalized construction. To ensure the success of our user science program, it will be important that we continually develop our beamlines and ensure that they remain in leading positions in their respective science and technical areas. Some of these enhancements are already planned over the next years, among which: (i) a second branch on the HIPPIE beamline dedicated to the research needs of the electrochemistry community, (ii) installation of a dedicated high-resolution powder diffraction setup, as well as a single-crystal diffraction side station for small molecules at DanMAX, (iii) the second branch of SoftiMAX dedicated toward the use of soft X-ray coherent diffraction imaging, (iv) the addition of an imaging endstation at NanoMAX with the goal to provide 10s of nanometer hard X-ray beams for nanotomography and ptychography.

Benefiting from the lessons learned from the tremendous progress in some beamline instrumentation developments at other lightsources will also be important. We have, for example, observed ever-increasing needs for adapted and modern data analysis tools to fully take advantage of the measured data from our beamlines. Like other light sources, we provide targeted data analysis support and temporary data storage. At present, our data policy states that we ensure that data remain available for a minimum of 7 years. We are also continuously evaluating the needs of the user community and adapting our data analysis strategy to maximize the scientific output of our facility.

Over the past decade, we have observed a revolution in the specifications and availability of some X-ray optical components that are key to the boost in the performance of many beamlines. This has been the case for focusing optics, for which the use of refractive [79] and diffractive optics has become widespread, but also thanks to the commercial availability of meter-long X-ray mirrors nearing atomic perfection [80]. We have also seen a similar boost in X-ray area detectors that have transformed the landscape of possibilities for performing advanced X-ray experiments. In tandem with developments in efficient beamline controls, data reduction pipelines, modern visualization, and data analysis tools, this creates exciting and transformational opportunities for developing experimental approaches that can impact more science areas.

Recently, we have also observed some shifts in the user community needs, as compared to decades-long of experience in X-ray experiments. Until recently, beamlines have universally excelled in doing one type of X-ray measurement remarkably well, targeting one specific scientific area/domain. However, an increasing number of user communities are interested in experimental approaches that allow them to better understand their samples by correlating spectroscopic, diffraction and scattering, and/or imaging data. These so-called “multi-modal” approaches are and continue to be an ever-increasing request for facilities that can accommodate such studies and allow to bring new perspectives in in situ and operando scientific questions.

From 2020 to 2022, our everyday lives across the globe were severely impacted by the SARS-CoV-2 pandemic. Lightsources worldwide also had to adapt during these troubled times. This fact forced many of us to rethink our approaches whenever possible to perform some of our experiments at synchrotron sources. Whereas remote users and remote access to beamlines have been in place for decades, principally in the area of Life Science at macromolecular crystallography beamlines across the globe, there were comparatively few opportunities at beamlines serving other science areas. The pandemic pushed the community of users and beamline scientists to innovate in some areas, which led to the remote operation of beamlines that previously and historically never offered this access mode as an option.

It will be important to continue to reflect on these lessons learned while we modernize some of our ways of approaching our user program to remain competitive and serve to the best of our abilities its current and future research needs.

## Opportunities and challenges

Over the next decade, MAX IV aims to continue operating and developing its 16 beamlines in addition to extending the instrumentalization of our storage rings with more beamlines. This will be achieved by continue strengthening the engagement of our ever-growing user community in close relationships with our stakeholders and funders. By doing so, we intend to continue being the synchrotron source of choice, principally serving the Nordic and Baltic regions’ current and future research needs. We expect that our existing and future beamlines will support a broad and diverse user science program, that will benefit society.

As mentioned, our two storage rings could accommodate up to 14 more beamlines. As part of our strategic process, we recently solicited input from the user community by gathering Expressions of Interest for new beamlines and major instrumentation. After an external review, these were translated into a prioritized list of beamlines that we will continue developing while actively looking for funding opportunities in partnership with the user community and our stakeholders. We anticipate repeating this process with a periodicity of two years.

In 2023 and for the first time, MAX IV is transitioning from a construction to an operation phase, i.e., all beamline construction projects are completed. It will therefore be important for the facility to strengthen its user science program by proactively maintaining and developing all aspects of our beamlines at the state-of-the-art, including undulators, optical components, X-ray and electron detectors, sample environments, controls and data systems, data reduction, analysis and visualization tools. These technical advancements are an integral part of optimizing the overall user experience. MAX IV strives to continue facilitating opportunities for multi-modal and integrative approaches, and strengthening partnerships with national and international scientific institutions, projects, training, and outreach activities.

The success of our beamlines will depend on our accelerators’ reliability, stability, and competitiveness to support our ambitions. This should ensure that our existing and future beamlines can fully contribute to provide an understanding of the atomic, molecular, long- and short-range, electronic and magnetic structure, and dynamics of equilibrium and non-equilibrium matter. Below are some opportunities that MAX IV will contribute to:

- MAX IV is in a privileged position to offer a unique platform for X-ray Photoelectron Spectroscopy that fully exploits the brightness of our two storage rings from the VUV to the soft, tender, and hard X-ray regime. This will open up transformational opportunities to probe in situ and operando surface and interface information of catalytic or electrochemical processes.
- We anticipate major developments in 2- and 3-dimensional imaging across the entire wavelength range available at MAX IV. We expect a democratization of imaging techniques at most 4th generation light sources thanks to their unprecedented small divergence and large degree of coherence of their X-rays. This translates into unprecedented performance of beamlines providing coherent X-ray diffraction, ptychographic, and tomographic imaging but also nano- and micro-probe spectro-diffraction microscopy techniques.
- The available photon flux will support the development of imaging techniques in the time-resolve domain, providing opportunities to probe the dynamics and kinetics of matter. These areas are very relevant for in situ and operando studies.
- Access to such small, intense, and coherent beams also creates opportunities to understand the structure of small samples but also heterogeneous, imperfect, soft, or weakly scattering matter.
- The unprecedented X-ray brightness of our small emittance 3 GeV storage rings offers new opportunities to further explore X-ray coherence on the entire wavelength spectrum, especially in the soft X-ray region (i.e., below 1 keV) where the beams are nearly diffraction limited. Similarly, these unique properties allow for exploring the use of the orbital angular momentum of light in the X-ray regime.

## Data Availability

No data associated with the manuscript.

## Footnotes

1 MAX stands for “Microtron Accelerator for X-rays” [2].

2 Aarhus University [13], Copenhagen University [14], Technical University of Denmark [15].

3 Chalmers University of Technology [18], University of Gothenburg [19], Karlstad University [20], Karolinska Institutet (KI) [21], The Royal Institute of Technology (KTH) [22], Linköping University [23], Linnæus University [24], Luleå University of Technology [25], Lund University [1], Malmö University [26], Swedish University of Agricultural Science [27], Stockholm University [28], Umeå University [29], Uppsala University [30].

4 Å. Andersson, L. Malmgren, and P.F. Tavares, On the possibilities to elongate bunches up to a factor of 10, by means of Higher Harmonic Cavities (HHC), MAX IV Internal note (2015).
